# Quantitative characterization of the urine and serum metabolomes of children is essential for ‘omics’ studies

**DOI:** 10.1186/s12916-018-1219-z

**Published:** 2018-11-26

**Authors:** Alicia DiBattista, Pranesh Chakraborty

**Affiliations:** 1 Newborn Screening Ontario, Children’s Hospital of Eastern Ontario, Ottawa, K1H 8L1 Canada; 2 Department of Pediatrics, Children’s Hospital of Eastern Ontario, Ottawa, K1H 8L1 Canada

**Keywords:** Metabolomics, Children, Metabolic profiling, LC-MS, NMR, Exposomics, Metabolome, Serum, Urine

## Abstract

Understanding the long-term health impacts of the early-life exposome requires the characterization and assimilation of multi ‘omics’ data to ultimately link molecular changes to exposures. In this way, markers associated with negative health outcomes, such as increased disease risk, can be ascertained. However, determining the extent and direction of metabolic perturbations relies on comparisons to existing metabolomic reference profiles. While such resources are increasingly available for adult populations, analogous tools for children are decidedly lacking. Lau et al. have compiled robust, translatable quantitative metabolomics data on urine and serum samples for European children across six study locations. Metabolites were associated with body mass index, diet and demographics, and correlated within and between biofluids. As a result, a novel association between urinary 4-deoxyerythronic acid and body mass index was uncovered. This work serves as a crucial reference for future studies in exposomics, and – more broadly – represents a significant step forward for metabolomics by creating the foundation for a comprehensive reference metabolome for children.

Please see related article: https://bmcmedicine.biomedcentral.com/articles/10.1186/s12916-018-1190-8

## Background

In their recent *BMC Medicine* article, Lau et al. [1] characterized the serum and urine metabolic profiles of European children, and determined metabolites associated with age, sex, body mass index (BMI), and dietary intake. This study was part of HELIX (Human Early-LIfe EXposome), a collaborative project between six longitudinal birth cohort studies that aims to characterize early-life physical and chemical exposures, and understand their impacts on growth, neurodevelopment, and respiratory health [2]. The exposome is conceptualized as the sum total of interrelated general exposures (e.g., geographical location, socioeconomic status) and specific external (e.g., pollution, diet) and internal (e.g., gut microflora, metabolism) exposures, and therefore represents non-genetic factors that can contribute to human disease [3]. As stated by the authors, researchers hypothesize that exposures during crucial developmental periods, such as periconception, the prenatal period, and early infancy can impact the predisposition to disease in the future [4]. Studies on the developmental origins of health and disease (DOHaD) therefore seek to characterize the detectable biomarkers that differentiate health from disease, and to correlate them to known exposures in order to contextualize and complement genomic data [5].

Metabolic syndrome is a collection of features, including obesity, hyperglycemia, and hypertension, which increase the risk of developing cardiovascular disease and type 2 diabetes (T2D) [6, 7]. Obesity rates are dramatically increasing in children, and it is estimated that, by 2025, 70 million children worldwide will be overweight or obese [8]. Understanding the association of exposures such maternal health, urban or rural environment, pollutants and dietary intake with ‘omics’ data can help to illuminate the impact of these early-life exposures on later-life health outcomes.

## Establishing a reference metabolome for future ‘omics’ studies

To define a set of metabolic markers that reflects the exposome first requires a high quality reference metabolome to be established. This was the primary objective of Lau et al. [1]. Such a reference is an invaluable tool for research because it facilitates further ‘omics' research. Characterizing baseline levels of metabolites in biofluids, and their associations with age and sex, provides a standard against which changes related to the exposome can be compared, thus helping to elucidate underlying biological mechanisms. For example, urine metabolomics has been successfully applied to assess adherence in adults to two complex dietary patterns, revealing markers capable of distinguishing between the diets with acceptable predictive accuracy [9]. While studies such as KarMeN [10], Husermet [11], the Human Serum Metabolome [12], the Human Urine Metabolome [13] and the human plasma metabolome [14] have established reference populations of various sizes, few comparable references existed for children [15].

Recent work confirming diagnoses of various inborn errors of metabolism highlights the importance of a quantitative reference metabolome – not only for determining pathological elevations in target analytes, but also for untargeted approaches that aim to discover additional or improved metabolic markers [16]. The use of the widely popular Biocrates Absolute*IDQ* p180 kit, along with appropriately stringent inclusion criteria, generated reproducible quantitative information for 177 serum metabolites. While this is by no means a comprehensive characterization of the either metabolome (which would require multiple analytical platforms and multiple sample preparations), data from the Biocrates kit are highly precise, reproducible, and – importantly – translatable between laboratories and instruments [17].

Generating robust, high-quality quantitative data for both the urine and serum metabolomes was a prudent choice for Lau et al.; many differentiating metabolites from biomarker discovery studies remain unidentified. They cannot be quantified for use in a targeted clinical assay, or be linked to metabolic pathways, genomic or proteomic data [18], and thus currently represent metabolic dead-ends. By further providing correlations both within and between the urine and serum metabolomes, Lau et al. [1] have demonstrated that while the serum metabolome reflects a greater degree of biological variation, these biofluids are complementary, and the urine metabolome may be of interest in future exposome studies, such as those involving dietary interventions or alterations in the microbiome. Finally, their discovery of a novel association between urinary 4-deoxyerythronic acid and BMI suggests that future studies on obesity and the onset of comorbidities such as T2D could target metabolites within the threonine metabolic pathway.

## Conclusion

This study by Lau et al. [1] represents a formidable effort in defining the serum and urine metabolomes of children. Metabolite concentration ranges encompassing a broad variety of compound classes and associations with age, sex, BMI and dietary intake have now been described, which serves as a precious resource for future metabolic studies in children. The broader metabolomics community can and should build out from this study by performing additional untargeted analyses utilizing complementary analytical platforms. The discovery of the novel association between 4-deoxyerythronic acid and BMI is an encouraging finding, and suggests that further studies on the HELIX subcohort may reveal interesting markers of early-life exposure.

## Abbreviations

BMI: Body mass index
DOHaD: Developmental origins of health and disease
HELIX: Human early-life exposome
Husermet: Human serum metabolome
KarMeN: Karlsruhe Metabolomics and Nutrition Study
T2D: Type 2 diabetes
